# Multidimensional analysis of adult patients’ care trajectories before a first diagnosis of schizophrenia

**DOI:** 10.1038/s41537-022-00256-6

**Published:** 2022-05-19

**Authors:** Alain Vanasse, Josiane Courteau, Mireille Courteau, Marc-André Roy, Emmanuel Stip, Marie-Josée Fleury, Alain Lesage, Sébastien Brodeur

**Affiliations:** 1 Département de médecine de famille et de médecine d’urgence, Université de Sherbrooke, Sherbrooke, QC Canada; 2 Groupe de recherche PRIMUS, Centre de recherche du Centre hospitalier universitaire de Sherbrooke (CRCHUS), Sherbrooke, QC Canada; 3 Département de Psychiatrie et Neurosciences, Université Laval, Québec, QC Canada; 4 Centre de Recherche CERVO, Québec, QC Canada; 5 Département de Psychiatrie et d’Addictologie, Université de Montréal, Montréal, QC Canada; 6 Department of Psychiatry and Behavioral Science, College of Medicine and Health Science, United Arab Emirates University, Al Ain, United Arab Emirates; 7 Department of Psychiatry, McGill University, Montréal, QC Canada; 8 Research Centre, Institut universitaire en santé mentale de Montréal (IUSMM), Montréal, QC Canada

**Keywords:** Schizophrenia, Psychosis

## Abstract

For patients at high-risk for developing schizophrenia, a delayed diagnosis could be affected, among many reasons, by their patterns of healthcare use. This study aims to describe and generate a typology of patients’ care trajectories (CTs) in the 2 years preceding a first diagnosis of schizophrenia, over a medico-administrative database of 3712 adults with a first diagnosis between April 2014 and March 2015 in Quebec, Canada. This study applied a multidimensional approach of State Sequence Analysis, considering together sequences of patients’ diagnoses, care settings and care providers. Five types of distinct CTs have emerged from this data-driven analysis: The type 1, shared by 77.6% of patients, predominantly younger men, shows that this group sought little healthcare, among which 17.5% had no healthcare contact for mental disorders. These individuals might benefit from improved promotion and prevention of mental healthcare at the community level. The types 2, 3 and 4, with higher occurrence of mental disorder diagnoses, represent together 19.5% of the study cohort, mostly middle-aged and women. These CTs, although displaying roughly similar profiles of mental disorders, revealed very dissimilar sequences and levels of care providers encounters, primary and specialized care use, and hospitalizations. Surprisingly, patients of these CTs had few consultations with general practitioners. An increased attentiveness for middle-aged patients and women with high healthcare use for mental disorders could help to reduce delayed diagnosis of schizophrenia. This calls for further consideration of healthcare services for severe mental illness beyond those offered to young adults.

## Introduction

Schizophrenia (SCZ) is among the most complex and severe mental disorders, associated with chronic psychotic symptoms, considerable disability, and a substantial healthcare use (HCU)^1–4^. The early identification of high-risk individuals is crucial to reduce the time gap between first psychotic symptoms and the initiation of adequate treatments^5–11^. Studies reported that brief attenuated psychotic symptoms appear in the “Late at-risk of psychosis state”^7,12–14^. However, since most of symptoms are non-specific, high-risk individuals remain difficult to detect, and the disease might go unnoticed for a period estimated at 1–2 years, even after the onset of diagnosable symptoms^3,5,7,12,15,16^.

High-risk individuals may also have comorbid conditions, such as anxiety, mood disorders, and substance use disorders. Although the vast majority of people with mental disorders do not seek healthcare for mental reasons, patients usually have previous contacts with healthcare services before a formal diagnosis of SCZ^17,18^. Thus, healthcare professionals may have opportunities to early identify patients at high risk and ensure rapid access to multidisciplinary approach and coordinated healthcare services and appropriate treatments^5,8–11,13,19,20^.

Healthcare-seeking behaviors for mental illness, throughout care settings and care providers, may be influenced by many factors at individual, environmental, and healthcare system levels. A better understanding of patients’ care trajectories (CTs) before a first diagnosis of SCZ could provide important knowledge on HCU and healthcare-seeking behaviors, that could affect delayed diagnosis and intervention. However, published studies focused largely on HCU following the diagnosis of SCZ and to date, available information before the diagnosis is fragmented and remains limited^9,21,22^.

For a comprehensive analysis of CTs, many distinct dimensions related to HCU should be considered, as proposed by the ‘6W’ multidimensional model of CTs^23^: Patients, with their individual attributes (“who”), responding to their illness conditions (“why”), may seek healthcare from different types of care providers (“which”) at different care settings (“where”), receiving treatments and therapy (“what”) over specific periods of time (“when”). Taking into account these multiple dimensions, the analysis of CTs is an ambitious task. In the last decade, the State Sequence Analysis (SSA) has become an efficient method for the analysis and visualization of longitudinal sequential data in the field of health service^24–30^. Using a SSA approach, a CT consists of a sequence of successive categorical states and transitions, each corresponding to a patient’s record of HCU at a given time. Accordingly, to achieve a comprehensive analysis of CTs, while avoiding complex sequences, CTs could be analyzed by partitioning HCU into multiple interconnected dimensions, considering the “who, why, where, which, what and when”^23,28^.

Analyzing CTs prior to a diagnosis of SCZ using observational studies could provide valuable empirical evidence on patients’ HCU, such as primary and specialized care services use for mental disorders, care settings and key care providers. This could benefit data-driven decision-making for organizational and clinical practices, as insights on healthcare-seeking behaviors for high-risk patients. This study postulates that analyzing CTs could provide valuable information on patterns of patients’ interactions with the healthcare system before a first diagnosis of SCZ, and that specific groups of patients could share similar patterns. Following this assumption, and according to the “late high-risk” phase and potential diagnosable symptoms, the purpose of this study is to describe patients’ CTs 2 years before a first diagnosis of SCZ and to propose a typology of CTs.

## Results

**Fig. 1:**
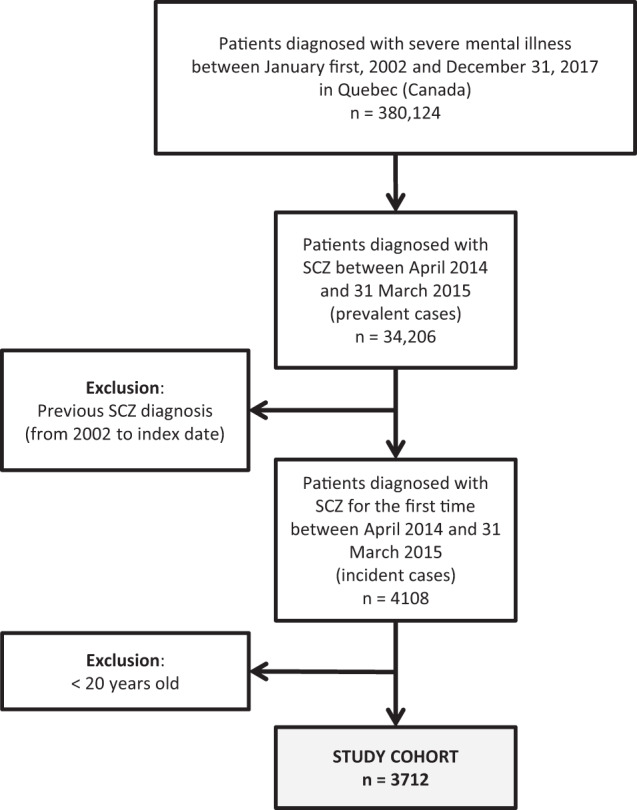
Study cohort flow diagram.

A specific cohort of 34,206 patients identified with SCZ during the year 2014–2015 was extracted from the SMD cohort. Excluding from the prevalent pool all patients that received a previous diagnosis of SCZ (from 2002 to index date), 4108 patients were considered as incident cases among which 3712 were aged 20 years or older (Fig. 1), 1934 (52.1%) were men and 1778 (47.9) women. The age distribution of the study cohort by sex is presented in Table 1 and shows that men were diagnosed earlier (mean age 42.2 years; standard deviation 17.5) than women (mean age 51.7 years; standard deviation 18.6). In addition to age, some characteristics differ significantly between sexes: compared to men, women had more comorbidities and were more likely of being diagnosed at an outpatient or a private clinic rather than at a hospital or an emergency department, but men were more likely to have a previous diagnosis of alcohol or drug abuse. Furthermore, only 102 (2.8%) patients had no previous all-cause HCU in the 2-year period before a SCZ diagnosis and 534 (14.4%) had no previous contacts for a mental disorder, men being more likely than women of not having such contacts with the healthcare system (Table 1).

**Table 1:** Characteristics of the study population by sex (*n* = 3712).

Characteristics^a^	Total (*n* = 3712)	Women (*n* = 1778)	Men (*n* = 1934)	*p* value
Age, median (IQR)	45 (30–60)	51 (36–66)	39 (26–55)	<0.0001
Age group, *n* (%)				<0.0001
20–34	1187 (32.0)	397 (22.3)	790 (40.8)	
35–54	1234 (33.2)	591 (33.2)	643 (33.2)	
55–64	559 (15.1)	315 (17.7)	244 (12.6)	
≥65	732 (19.7)	475 (26.7)	257 (13.3)	
PPDIP status, *n* (%)				0.5239
Not admissible	939 (25.3)	451 (25.4)	488 (25.2)	
Admissible—regular	1125 (30.3)	553 (31.1)	572 (29.6)	
Admissible—LRFA/GIS	1648 (44.4)	774 (43.5)	874 (45.2)	
Material deprivation quartiles, *n* (%)^b^				0.4509
1	629 (19.6)	299 (19.4)	330 (19.9)	
2–3	1558 (48.6)	768 (49.7)	790 (47.6)	
4	1018 (31.8)	477 (30.9)	541 (32.6)	
Social deprivation, *n* (%)^a^				0.0338
1	570 (17.8)	258 (16.7)	312 (18.8)	
2–3	1434 (44.7)	673 (43.6)	761 (45.8)	
4	1201 (37.5)	613 (39.7)	588 (35.4)	
Rurality, *n* (%)^c^				0.1479
Metropolitan	2645 (74.3)	1304 (75.5)	1341 (73.1)	
Small town	409 (11.5)	197 (11.4)	212 (11.6)	
Rural	507 (14.2)	226 (13.1)	281 (15.3)	
First Dx: Care setting, *n* (%)				<0.0001
Hospital	1525 (41.1)	718 (40.4)	807 (41.7)	
ED	1006 (27.1)	431 (24.2)	575 (29.7)	
Outpatient clinic	596 (16.1)	318 (17.9)	278 (14.4)	
Primary care clinic/Other	585 (15.8)	311 (17.5)	274 (14.2)	
First Dx: Physician specialty, *n* (%)				0.0137
Psychiatrist	1814 (48.9)	882 (49.6)	932 (48.2)	
GP	955 (25.7)	460 (25.9)	495 (25.6)	
Other specialities	58 (1.6)	38 (2.1)	20 (1.0)	
Unspecified^d^	885 (23.8)	398 (22.4)	487 (25.2)	
Alcohol abuse, *n* (%)	704 (19.0)	289 (16.2)	415 (21.5)	<0.0001
Drug abuse, *n* (%)	863 (23.2)	342 (19.2)	521 (26.9)	<0.0001
Comorbidity index, *n* (%)				<0.0001
0	2569 (69.2)	1125 (63.3)	1444 (74.7)	
1–2	588 (15.8)	322 (18.1)	266 (13.8)	
≥3	555 (15.0)	331 (18.6)	224 (11.6)	
At least one HCU 2 years before index date, *n* (%)	3610 (97.3)	1753 (98.6)	1857 (96.0)	<0.0001
At least one mental HCU 2 years before index date, *n* (%)	3178 (85.6)	1566 (88.1)	1612 (83.4)	<0.0001

^a^Unless otherwise specified, there are no missing values.

^b^Missing values: *n* = 507.

^c^Missing values: *n* = 151.

^d^Unspecified: missing specialty occurs mainly during a hospitalization.

**Fig. 2:**
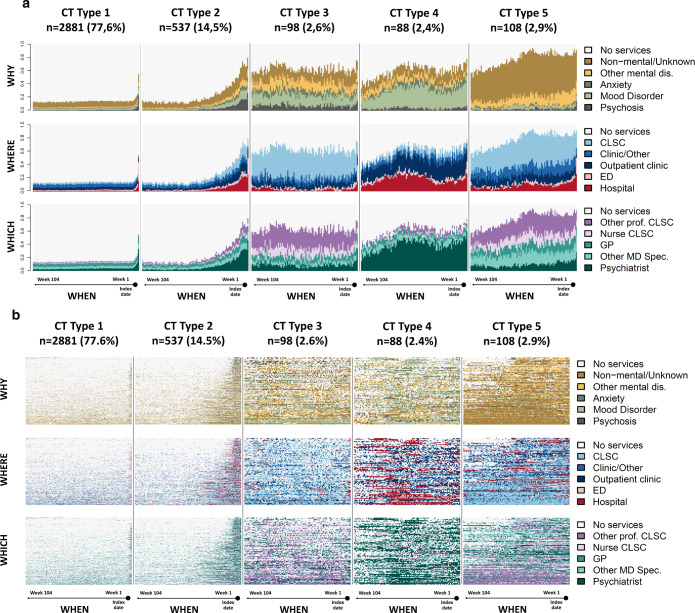
State distribution plots (**a**) and sequence index plots (**b**), of the typology of care trajectories (CTs) by dimension (why, where and which), using simple Hamming as the distance measure and HCA as the clustering method. State distribution plots show the distribution of states (proportion) for each time unit point (104 weeks) prior the first diagnosis of SCZ. In sequence index plots, each line represents an individual’s CT sequence.

**Fig. 3:**
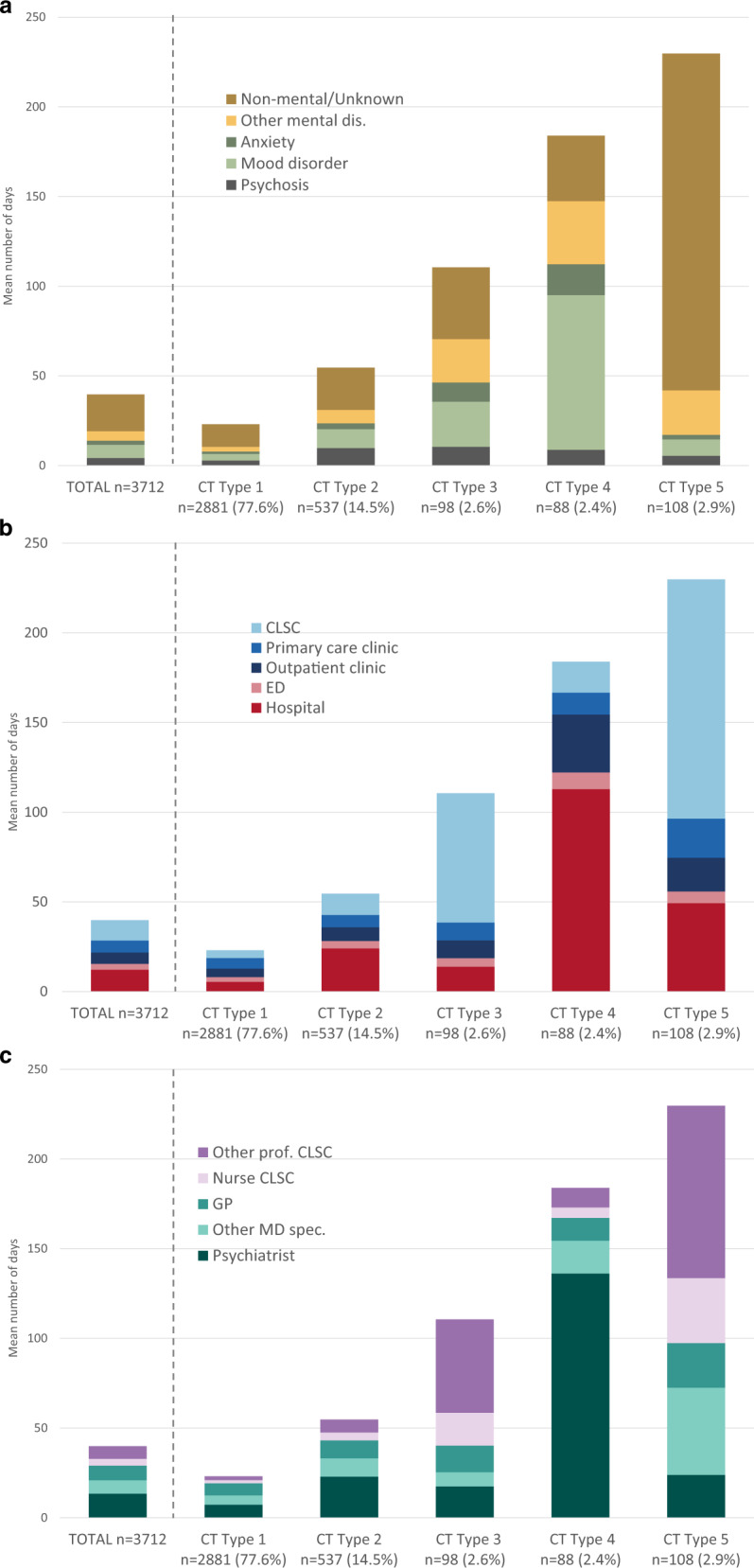
Mean number of days of HCU according to the **a** reason for consultation, **b** care setting and **c** care provider 2 years before index date.

In the multidimensional SSA analyses, patients with “similar” CTs were classified according to five distinct and homogeneous CT types (the typology of CTs). Figure 2a, b presents the state distribution plots and the sequence index plots, respectively, for the five types of CTs according to dimensions “why”, “where” and “which”. The former plots (Fig. 2a) present, for each of the 104 weeks (*x*-axis), the proportion of patients in each state (*y*-axis), while the latter plots (Fig. 2b) present the patient’s state sequence over the 2 years, i.e., each line represents an individual CT sequence over time. Priorities among states in a patient’s record of HCU at a given week need to be established a priori to perform SSA. The mean number of days in each state as well as the proportion of patients using the different HCU by CT type and dimension, presented in Fig. 3 and Supplementary Table 1 respectively, offer a complementary view of the HCU that helps to interpret the typology of CTs. Table 2 presents patients’ characteristics associated with each type of CTs.

**Table 2:** Characteristics of the study cohort by the typology of care trajectories (CTs) (*n* = 3712).

Characteristics^a^	Total	CT Type 1	CT Type 2	CT Type 3	CT Type 4	CT Type 5	*p v*alue
		Low HCU	Sharp 12-month HCU increase, mental dis.	Mod. HCU, mental dis., primary care, community services	High HCU, mental dis., specialized care, psychiatrists	High HCU, Non-mental dis.	
	*n* = 3712	n = 2881 (77.6%)	*n* = 537 (14.5%)	*n* = 98 (2.6%)	*n* = 88 (2.4%)	*n* = 108 (2.9%)	
Age, median (IQR)	45 (30–60)	44 (29–58)	47 (32–64)	55 (42–68)	46 (34–66)	69 (55–81)	<0.0001
Age group, *n* (%)							<0.0001
20–34	1187 (32.0)	977 (33.9)	159 (29.6)	16 (16.3)	23 (26.1)	12 (11.1)	
35–54	1234 (33.2)	996 (34.6)	167 (31.1)	32 (32.6)	25 (28.4)	14 (13.0)	
55–64	559 (15.1)	427 (14.8)	81 (15.1)	19 (19.4)	15 (17.1)	17 (15.7)	
≥65	732 (19.7)	481 (16.7)	130 (24.2)	31 (31.6)	25 (28.4)	65 (60.2)	
Sex, *n* (%)							<0.0001
F	1778 (47.9)	1296 (45.0)	282 (52.5)	70 (71.4)	59 (67.0)	71 (65.7)	
M	1934 (52.1)	1585 (55.0)	255 (47.5)	28 (28.6)	29 (33.0)	37 (34.3)	
PPDIP status, *n* (%)							<0.0001
Not admissible	939 (25.3)	756 (26.2)	133 (24.8)	10 (10.2)	26 (29.6)	14 (13.0)	
Admissible—regular	1125 (30.3)	889 (30.9)	161 (30.0)	19 (19.4)	29 (33.0)	27 (25.0)	
Admissible—LRFA/GIS	1648 (44.4)	1236 (42.9)	243 (45.2)	69 (70.4)	33 (37.5)	67 (62.0)	
Material deprivation quartiles, *n* (%)^b^							0.0943
1 (less deprived)	629 (19.6)	478 (19.4)	103 (21.5)	8 (8.9)	22 (27.2)	18 (19.2)	
2–3	1558 (48.6)	1190 (48.4)	231 (48.2)	49 (54.4)	42 (51.8)	46 (48.9)	
4 (most deprived)	1018 (31.8)	793 (32.2)	145 (30.3)	33 (36.7)	17 (21.0)	30 (31.9)	
Social deprivation quartiles, *n* (%)^b^							0.0396
1 (less deprived)	570 (17.8)	453 (18.4)	87 (18.2)	10 (11.1)	12 (14.8)	8 (8.5)	
2–3	1434 (44.7)	1084 (44.0)	227 (47.4)	39 (43.3)	43 (53.1)	41 (43.6)	
4 (most deprived)	1201 (37.5)	924 (37.6)	165 (34.4)	41 (45.6)	26 (32.1)	45 (47.9)	
Rurality, *n* (%)^c^							0.0370
Metropolitan	2645 (74.3)	2072 (75.3)	372 (71.1)	63 (65.0)	66 (75.9)	72 (69.9)	
Small town	409 (11.5)	294 (10.7)	75 (14.3)	12 (12.4)	10 (11.5)	18 (17.5)	
Rural	507 (14.2)	385 (14.0)	76 (14.5)	22 (22.7)	11 (12.6)	13 (12.6)	
First Dx: Care setting, *n* (%)							<0.0001
Hospital	1525 (41.1)	1135 (39.4)	243 (45.2)	39 (39.8)	40 (45.4)	68 (63.0)	
ED	1006 (27.1)	856 (29.7)	109 (20.3)	15 (15.3)	14 (15.9)	12 (11.1)	
Outpatient clinic	596 (16.1)	418 (14.5)	117 (21.8)	22 (22.4)	27 (30.7)	12 (11.1)	
Primary care clinic/Other	585 (15.8)	472 (16.4)	68 (12.7)	22 (22.4)	7 (8.0)	16 (14.8)	
First Dx: Physician specialty, *n* (%)							<0.0001
Psychiatrist	1814 (48.9)	1379 (47.9)	294 (54.8)	44 (44.9)	60 (68.2)	37 (34.3)	
GP	955 (25.7)	769 (26.7)	118 (22.0)	29 (29.6)	13 (14.8)	26 (24.1)	
Other specialist/Unspecified^d^	943 (25.4)	733 (25.4)	125 (23.3)	25 (25.5)	15 (17.0)	45 (41.7)	
Alcohol abuse, *n* (%)	704 (19.0)	524 (18.2)	136 (25.3)	10 (10.2)	17 (19.3)	17 (15.7)	0.0003
Drug abuse, *n* (%)	863 (23.2)	641 (22.2)	162 (30.2)	15 (15.3)	26 (29.6)	19 (17.6)	0.0001
Comorbidity index, *n* (%)							<0.0001
0	2569 (69.2)	2147 (74.5)	326 (60.7)	45 (45.9)	34 (38.6)	17 (15.7)	
1–2	588 (15.8)	445 (15.4)	76 (14.2)	27 (27.6)	20 (22.7)	20 (18.5)	
≥3	555 (15.0)	289 (10.0)	135 (25.1)	26 (26.5)	34 (38.6)	71 (66.7)	

^a^Unless otherwise specified, there are no missing values.

^b^Missing values: *n* = 507.

^c^Missing values: *n* = 151.

^d^Unspecified: missing specialty occurs mainly during a hospitalization.

The most common type, CT Type 1 (*n* = 2881, 77.6%), can be described as low HCU with no predominant diagnosis, care settings or care providers (Figs. 2 and 3). Almost 18% of patients in this group did not have previous registered contact with a mental disorder diagnosis during the 2 years before the SCZ diagnosis (Supplementary Table 1). Patients associated with this type can be characterized as younger patients, mainly male, with fewer comorbidities (Table 2). The second type in frequency is Type 2 (*n* = 537, 14.5%) and can be described as low HCU for non-mental or unspecified reasons at the beginning of the observation period followed by a sharp increase of HCU 12 months before their first diagnosis of SCZ, mainly for psychosis and mood disorders in hospital settings, and involved psychiatrists (Figs. 2 and 3 and Supplementary Table 1). Patients in Type 2 are mainly middle-aged patients with a high proportion of alcohol and drug abuse (Table 2). The remaining three other types of CTs are less frequent but constitute intense HCU. Different patterns of HCU emerged: Type 3 (*n* = 98, 2.6%) can be described as moderate HCU for various mental disorders mainly in community services (CLSCs), with care delivered mostly by professionals and nurses (Figs. 2 and 3). Patients in Type 3 are mainly older women, living in small towns or rural areas in a higher proportion than the other groups, with a lower socioeconomic status and a high comorbidity index (Table 2); Type 4 (*n* = 88, 2.4%) can be described as high HCU mostly for mood disorders in hospitals and outpatient clinics, with frequent psychiatrists’ encounters (Figs. 2 and 3). Patients in Type 4 are mostly middle-aged women, less deprived, with a high proportion of alcohol and drug abuse and a high comorbidity index (Table 2); Finally, Type 5 (*n* = 108, 2.9%) can be described as high HCU, mainly for non-mental reasons, with a large proportion seen in hospital and CLSCs, and seen by non-psychiatrists (Figs. 2 and 3 and Supplementary Table 1). Patients in Type 5 are mostly older female, with low socioeconomic status, living in more socially deprived areas, and with important comorbidities.

### Sensitivity analyses

To test the robustness of the results, four additional SSA were performed with different distance measures or clustering methods: HCA method with dynamic Hamming (Supplementary Fig. 2), generalized Hamming (Supplementary Fig. 3) and optimal matching (Supplementary Fig. 4) distances, and K-means clustering method with simple Hamming distance (Supplementary Fig. 5). All three SSA using Hamming distances produced essentially a similar typology of CTs, although the distribution amongst group was slightly different. Results using optimal matching (Supplementary Fig. 4) were slightly different than those obtained with the simple Hamming distance: shared by more than 92.9% of patients, two important types of CTs emerged (types 1 and 2), showing low and moderate HCU and a mixture of diagnoses, care settings and care providers. Type 2 shows a progressive non-exponential increase of HCU for mental disorders. However, two types of HCU for mental conditions also emerged (types 3 and 4), similar to those observed in the main simple Hamming SSA. Changing the clustering methods to k-means produced a totally different and less informative typology of CTs, with CT types differing only in terms of HCU intensity (from very low to very high HCU), all with a mixture of reasons for consultation, care settings and care providers (Supplementary Fig. 5). Changing the priority order of states associated with the care providers (with GP being prioritized over other MD specialist) produced essentially the same results as the main SSA analysis (Supplementary Fig. 6). Finally, the choice of the time unit is crucial in SSA. Two other SSA analyses were performed using months (26 months prior to the SCZ diagnosis) and days as time units (728 days prior to the SCZ diagnosis). As observed in a previous study^28^, the analysis by months (Supplementary Fig. 7) produces results somewhat similar to those obtained by weeks), although less interesting because of the dilution of the information due to the priority assumption for the different states. Analyses by days (Supplementary Fig. 8) produced five groups, one with low HCU composed of 92.2% of patients, another 6.5% very similar to the CT Type 2, and three groups with very few patients (representing less than 0.13% of the study cohort).

## Discussion

Patients have been categorized into five distinct and homogenous types of CTs, providing a new perspective on observed variations of patients’ patterns of diagnoses and HCU before a first diagnosis of SCZ. Instead of following a prior assortment of patients (e.g., by age and sex), this SSA used a data-driven approach, where patients were entirely partitioned according to the dissimilarity measure of their sequences of HCU over time. Nonetheless, some significant variations emerged, mostly between sex and age groups. Results on the overall cohort show that men were on average diagnosed earlier than women, with mean age 42.2 years for men and 51.6 years for women, which is slightly higher than previous studies from Canada and other countries, although different in population^10,31–33^. Since patients of this cohort are adults aged 20 years and over, this explains, among other factors, the older age at diagnosis.

As expected, patients sought healthcare for various mental disorders occurring before the diagnosis of SCZ, such as mood disorders, anxiety and substance-induced or non-specific psychosis, as well as concomitant non-mental conditions^3,5,12,13,16,34^. The typology of CTs could raise some concerns, but also reveals indications for improving the access and quality of mental healthcare services, as well as potential windows of opportunities for early identification and intervention for SCZ and related disorders^6–9,11^.

First, the most shared pattern of HCU in the 2 years before a first diagnosis of SCZ—the CT Type 1—reveals that as much as 77.6% of the total cohort sought few healthcare, and about half of which was not associated with mental disorders. Although almost all patients of this group had at least one record of HCU, nearly one in five patients had no registered mental disorder diagnosis. As a result, at the level of the healthcare system, one could almost describe the CT Type 1 as a “low care seeking” group of patients at high risk for developing SCZ. As most patients of this cohort seek little healthcare, SCZ may be undetected until older age. Patients of this group were younger, more often males, had fewer comorbidities, and received their first diagnosis more frequently at an emergency department than patients of other CTs types. A previous study in Quebec has found that before the diagnosis of SCZ-spectrum psychosis in a population of 14–25 years old, 32% of cases had no healthcare contact for a mental disorder, and almost 50% received their diagnosis in the emergency department^22^. Various factors could influence healthcare-seeking behaviors. Some studies reported a deficit of knowledge about SCZ and other mental disorders, and significant stigmatized positions and beliefs about these diseases, in patients, their families and in the population^4,12,35^. Other studies have shown that most people with mental disorders had little or no contact with mental healthcare and, amongst other determinants of mental help-seek behaviors, middle-aged people and women were most likely to have contacts with mental healthcare^17,18,36^. Online help-seeking for mental health could also explain a portion of low HCU, especially for the younger population^37^. There are also potential barriers to mental health services use. In Canada, a populational survey revealed that the most common barrier was acceptability (e.g., no belief that healthcare could help, not knowing where to seek help, stigmatization), followed by availability (e.g., care providers not available when needed, waiting time too long) and accessibility (e.g., problems with costs, transport, schedule)^38^.

Second, the CTs Types 2, 3 and 4, labeled “Mental disorders” groups, while different in patterns of HCU, shared moderate to high levels of contacts with the healthcare system. These groups represent together almost 20% of the study cohort. Compared to the Type 1, patients of these groups were predominantly middle-aged female, more often diagnosed with mood disorders, particularly in Type 4, and with substance abuse in Types 2 and 4. Also, state distribution of care settings and care providers of Type 4 and Type 2 in the late 12 months, suggest that these patients experienced acute illness conditions (hospitalizations, outpatient clinics and psychiatrists). Findings also indicate age and gender inequity in CTs, which may lead to delayed diagnosis. Patients of these groups seem to represent older adults and women with late onset of SCZ, experiencing more frequently long illness courses such as anxiety and mood disorders, and possibly more often misdiagnosed with affective psychosis^10,39–42^.

Upon closer examination of state distribution plots (Fig. 3a) and sequence index plots (Fig. 3b), observed patterns of CTs Types 3 and 4, revealed interesting information. While State distribution plots show distribution and levels of HCU, sequence index plots enlighten the impressive complexity of patterns of diagnoses and HCU at individual levels. Sequence index visualization reveals that numerous patients experienced series of diagnoses of either mood disorders, psychosis, anxiety and other mental and non-mental disorders, through successive encounters with diverse categories of physicians and professional care providers, either at ambulatory or inpatient care settings. Moreover, numerous patients of the Type 4 experienced multiple episodes of hospitalization and/or long hospital stays and constant psychiatrist’s encounters, which suggests acute episodes of illness, while patients of the Type 3 undergone regular and numerous encounters with GPs, psychiatrists and healthcare professionals mostly at primary and community-based care settings. Yet, for those persisting “moderate and high mental HCU” groups of patients, SCZ remained undiagnosed by the healthcare system. Despite variations in the illness course in the pre-diagnosis phase, theoretically, “detectable” symptoms and manifestations of brief attenuated psychotic symptoms may appear in the “late at-risk of psychosis state”^5,7,12,13^. It has been reported also that the onset of SCZ happens after 40 years old for 20% of women^19,39–42^. Thus, results of the CT Types 2, 3 and 4 suggest potential windows of opportunities for earlier diagnosis and intervention. That being said, even after the manifestation of diagnosable symptoms, studies have shown that SCZ could remain unnoticed, and this for about 2–3 years^3,7,11–13,16^. Moreover, other studies have reported that healthcare resources utilization is often for non-mental reasons^34,43^, and comorbid conditions and somatic symptoms associated with common mental disorders could complicate healthcare-seeking^12,21,44^. Patients of Type 5, which are older and predominantly female, may represent those complex patients with multiple comorbid conditions and high HCU, mostly for non-mental diagnoses.

### Overall care trajectories

Severe mental disorders require coordinated care between primary and specialized healthcare services, involving general practitioners, psychiatrists, nurses, and other professionals^4,13,18–20^. Surprisingly, except for the labeled “non-mental disorders” CT Type 5, the results reveal that few encounters occurred with general practitioners and primary care providers, compared to specialists in inpatients and specialized care settings. Moreover, compared to specialized care, there were almost no changes in primary care use over time. Overall, the most common contacts for a first diagnosis of SCZ were psychiatrists (48.9%), followed by general practitioners (25.7%), at hospital and emergency departments. This could reflect the low care seeking among younger adults, a possible overuse of emergency departments and specialized care and consequently, the need to improve the access to mental health services in primary care^22,45^. Little is known about HCU for mental disorders preceding a specific diagnosis of SCZ and related disorders^9,21,22^. Few studies in similar healthcare systems, although different in populations and contexts, reported more contacts with GPs and a rise in primary care use several years before the diagnosis^46,47,48^. Nonetheless, these new findings on CTs are difficult to compare, since they result from a different methodological approach.

### Strengths and limitations

The strengths of this study are multiple. First, it uses an exhaustive longitudinal dataset of patients with severe mental disorders in Quebec, with linked medico-administrative data from multiple sources, such as patients’ demographic information, inpatients and outpatients, emergency and primary care medical records and community-based health services in Quebec, Canada. Second, its innovative multidimensional approach of SSA allows a comprehensive exploration of the most shared CTs, considering altogether patterns of diagnoses and reasons for consultations, care settings and care providers. The multidimensional approach for CT analysis also allows the possibility to include as much as 19 states, by partitioning HCU into three distinct sequence-dimensions, thus avoiding “overplotting” issues of complex sequences^49,50^. To our knowledge, this is the first study that proposes such a comprehensive perspective of CTs for SCZ, allowing an intuitive and straightforward examination of different types of patterns of HCU as a whole. This approach could be applicable for any other context and population, allowing comparisons of sequential patterns of HCU between similar healthcare systems.

Caution is needed when interpreting results based on administrative data, due to its inherent limitations: some important variables related to patients’ individual and environmental attributes, such as familial and social context at the individual level, which may significantly influence healthcare-seeking, are not available in administrative data. Furthermore, some diagnoses were grouped in the I-CLSC database, such as “mood disorders”, which include depression and bipolar disorders, and “psychosis”, which includes SCZ and other psychosis symptoms. For this specific problem, an examination revealed that amongst all psychosis diagnoses reported in the hospital discharge and medical services registers after the first diagnosis of SCZ, about 75% were SCZ. There are also potential limitations related to the choice of the distance measure between pairs of patients’ sequences of HCU. For example, the Hamming distance proceeds by a position-wise comparison and is thus very sensitive to timing mismatches. However, to test the robustness of the results, we performed several sensitivity analyses using different distance measures.

## Conclusion

For data-driven decision-making and clinical practice, this study has provided new knowledge on patients’ patterns of HCU 2 years before a first diagnosis of SCZ. Results confirm that groups of patients shared similar types of CTs, which may shed new light on windows of opportunities for the early detection and intervention for patients at high risk. Most people, especially young adults who sought little mental healthcare, might benefit from an improvement of promotion and prevention at the community level, such as awareness and acceptance of mental disorders, as well as prioritization of the accessibility to mental health services in primary care. The findings also highlight the importance of an increased attentiveness for middle-aged and older patients with mental disorders, as well as women who are often affected with different symptoms, and who might be disproportionately disadvantaged for early intervention by the lack of recognition at the healthcare system level^10,39,40^. Results also emphasis the importance of rapid referral to multidisciplinary assessments of early signs of SCZ and related disorders, including psychiatric history of prior diagnoses, hospitalizations and treatments^19,35,51^. There is obviously a need for more accurate sights on opportunities for earlier detection of SCZ and several studies using a multidimensional approach on CTs could be proposed. With a prior partition of patients by sex and by age (“who”), one could consider more detailed patterns of diagnoses in specialized care such as bipolar disorders and depression, and additional information such as accessibility to healthcare facilities (“where”), continuity of care and access to physicians (“which”), as well as treatments and follow-up (“what”).

## Methods

This study used a large longitudinal medico-administrative database of 380,124 patients diagnosed with severe mental disorders (including SCZ, bipolar disorder, and other psychosis). The analysis of CTs considered five of the six dimensions related to HCU^23^: patients’ attributes, e.g., age, sex, comorbidities (“who”), diagnosis or reason for HCU, e.g., depression, psychosis, SCZ or non-mental disorders (“why”), care settings, e.g., hospital, emergency department, primary care clinic (“where”), category of care providers, e.g., psychiatrist, general practitioner, nurse (“which”) and sequence of HCU over time (“when”). A modified multidimensional approach (Supplementary Fig. 1) of SSA previously developed^28^, was used to describe and classify patients’ CTs before a first diagnosis of SCZ.

### Design and data sources

Patients’ data for this population-based retrospective cohort study were acquired from the provincial health insurance board (Régie de l’assurance maladie du Québec (RAMQ)), which manages universal health insurance to Quebec residents, including coverage for physician and hospital services^52^. This universal health program is complemented by a public prescription drug insurance plan covering almost all people aged 65 and over, all recipients of last-resort financial assistance, and individuals who do not have access to a private drug insurance plan. The RAMQ manages administrative health registers including patients’ demographic information file, the hospital discharge register, the medical services database (containing information from physicians’ claims for services provided in outpatient clinics, emergency and primary care clinics), and since April 2012, the I-CLSC database, containing a wide range of primary care services provided at local community service centres (CLSC) by nurses, salaried physicians, nutritionists, physiotherapists, psychologists, and social workers. CLSCs are public health organizations, both financial in terms of governance^53^. The hospital discharge register contains information on hospitalizations’ date, length of stay, main and secondary diagnoses (ICD-9 before April 2006; ICD-10 thereafter). The patients’ demographic information file provides information on patients’ age, sex, geographical location of residency, and date of death. The medical services register provides the date of service, the location and the diagnosis (ICD-9) associated with the service provided. The validity of diagnoses recorded in the RAMQ medical services claims and hospital discharge databases have been assessed for a variety of chronic conditions^54,55^. The I-CLSC register^56^ provides information on the date of intervention, the reason for intervention (with its own coding system inspired by coding systems such as ICD-9, ICD-10 and DSM-V), and the type of professional encountered during a specific visit at CLSC. The I-CLSC database provides reliable data relating to the organization of care^57^. Using a unique encrypted identifier, patient data from these registers were linked to provide information on demographic, medical and HCU information. Neighborhood characteristics (rural/urban, material and social deprivation) were linked to patients’ data using their geographical location of residency included in the patients’ demographic information file.

### Studied population

Extracted from a larger cohort database on severe mental disorders (including SCZ, bipolar, and other psychosis disorders), the study cohort included all incident cases of SCZ aged 20 years and older diagnosed between April 1st, 2014 and March 31, 2015 (referred to as 2014–2015). Prevalent cases of SCZ were defined as patients receiving a diagnosis of SCZ and related disorders (ICD-9: 295; ICD-10: F20, F21, F23.2, F25) during a hospitalization or a medical visit in the year 2014–2015. To obtain incident cases only, we removed from the prevalent cases all patients with a previous diagnosis of SCZ 12 years prior index date^58^. The index date is the date of the first SCZ diagnosis during the 1-year period of 2014–2015. Since patients aged under 18 have access to adapted care and social services (youth centers, pediatrics), that could not be compared to adult care use^59^, we restricted the study population to adults (18 years and older) at the beginning of follow-up (2 years before the diagnosis of SCZ).

### Care trajectory variables

Defined as sequences of HCU over time, CTs were measured in a 2-year period (728 days) before a first diagnosis of SCZ. Connected with this temporal dimension (“When”), diagnoses (“Why”) were categorized as: psychosis (including alcohol and drug-induced psychosis) (ICD-9: 295, 297, 298, 291.3, 291.4, 291.5, 291.9, 292.1, 292.2, 292.8, 292.9; ICD-10: F20-F29, F10-F19 with 0.5, 0.7, 0.8, 0.9; and their corresponding I-CLSC codes), mood disorder (ICD-9: 296, 300.4, 311; ICD-10: F30-F39; and their corresponding I-CLSC codes), anxiety (ICD-9: 300 except 300.4; ICD-10: F40-F48; and their corresponding I-CLSC codes), other mental disorders (such as personality disorders, intellectual disability, neurocognitive disorders, etc.), non-mental disorders (other than ICD-9: 290–319; ICD-10: F00-F99; and their corresponding I-CLSC codes) or missing diagnosis; care settings (“Where”) were categorized as hospitals, emergency departments, outpatient clinics, primary care clinics and CLSCs; care providers (“Which”) were categorized as psychiatrists, other MD specialists, general practitioners, nurses in CLSC and other professionals in CLSC (e.g., social worker, psychologist, pharmacist, beneficiary attendant, etc.).

### Other variables

Characteristics of patients, as information related to the dimension “who”, included: age (continuous and categorical: 20–34, 35–54, 55–64, ≥65); sex (M, F); public prescription drug insurance plan status; and type of neighborhood (quartile of material and social deprivation; metropolitan, small town, rural); and a non-mental comorbidity index. The comorbidity index selected is proposed by Simard et al.^60^, which uses a combination of 31 conditions from the 17 Charlson’s and the 30 Elixhauser medical conditions^61,62^. This index was measured in the 2 years before the first diagnosis of SCZ and excludes mental disorders. The public prescription drug insurance plan admissibility status at the index date (as a proxy measure of low-income/unemployment status) includes four categories: not admissible (people with a private drug insurance plan); admissible and age ≥65 years with guaranteed income supplement; admissible and being a recipient of last-resort financial assistance; or regular recipient.

Other health care use variables not specifically used to define the CTs were also assessed as a description purpose: the place where the first SCZ diagnosis was registered (at a hospital with a principal diagnosis of SCZ, at a hospital with a secondary diagnosis of SCZ, during an emergency department visit, in an outpatient visit, in another clinic or place); the specialty of the healthcare provider reporting the first SCZ diagnosis (psychiatrist, other MD specialist, general practitioner, or unspecified in the case were the diagnosis was registered at the time of hospitalization); a diagnosed alcohol abuse in the previous 2 years (ICD-9: 291, 303, 305.0, 980.0, 980.1, 980.8, 980.9; ICD-10: F10, T51.0, T51.1, T51.8, T51.9); and a diagnosed drug abuse in the previous 2 years (ICD-9: 292, 304, 305, 965.0, 965.8, 967.0, 967.6, 967.8, 967.9, 969.4, 969.5, 969.6, 969.7, 969.8, 969.9, 970.8, 982.0, 982.8; ICD-10: F11-F19, F55, T40, T42.3, T42.4, T42.6, T42.7, T43.5, T43.6, T43.8, T43.9, T50.9, T52.8, T52.9).

### Handling of missing values

Contrary to survey data where missing values are customary, the variables derived from the administrative data have very few missing data. For example, the variables sex, age and public prescription drug insurance plan status have no missing values. Variables related to deprivation and rurality have however missing values, mainly because they result from a linkage between patients’ data and neighborhood data using the geographical location of residency included in the patients’ demographic information file as the linkage key. Missing values may occur because of a missing or incorrect geographical location of residency included in the patients’ demographic information file, or because there is missing information regarding material and social deprivation indices. Variables that use diagnoses reported in the database (such as alcohol and drug abuse and comorbidity index) are limited to the non-missing information on diagnoses. In the hospital discharge database, all hospitalization has at least one reported diagnosis, and as many as 95% have a reported diagnosis in the physician claims database. For these variables, if a specific medical condition is not reported in the database, it is assumed that the condition is not present.

### Statistical analysis

#### Measurement of CTs before a first diagnosis of SCZ

To characterize CTs and define homogeneous groups of CTs, we used a multidimensional version of a SSA^28^ (Supplementary Fig. 1). SSA was specifically developed and used to analyze sequential data^63^, particularly in social sciences.

For this study, CTs were measured in the 2-year period before the index date (first SCZ diagnosis) and “weeks” was chosen as the time units. For each week (“when”), we defined the following dimension-specific states: (1) The “Why” dimension was divided into six possible states with the following priority order: psychosis, mood disorder, anxiety, other mental disorder, non-mental disorders, and no HCU; (2) The “Where” dimension was divided into six possible states with the following priority order: hospitals, emergency departments, outpatient clinics, primary care or private clinics, CLSCs, and no HCU; (3) The “Which” dimension was divided into six possible states with the following priority order: psychiatrists, other MD specialists, general practitioners, nurses in CLSC, other professionals in CLSC, and no HCU. Then, for every patient, sequences of HCU were defined; one for the “why” dimension, one for the “where” dimension and one for the “which” dimension. If a patient has more than one state during a given unit of time (e.g., hospitalization and consultation in CLSC within the same week), the state with the highest priority, as listed above, was selected. The priority orders associated with each dimension were based on experts’ opinion, and established by the severity of medical events (e.g., hospitalization was prioritized over all other medical consultations). Also, as the specific focus of the study, mental disorders were prioritized over non-mental disorders. A distance matrix was calculated for each dimension containing the distance (proximity) between all pairs of patients’ CTs. The simple Hamming metric^64^ was used to measure the distances. A pooled distance matrix between CT sequences was defined as the sum of the three dimension-specific distances. This was done to propose a unique typology of CTs that accounts for all three dimensions.

#### Classification of CTs (typology of CTs)

Next, based on this pooled distance matrix, a hierarchical cluster analysis was used to classify patients with similar CTs^64,65^, In hierarchical cluster analysis, each patient starts in his own cluster, and then pairs of clusters are merged as one moves up the hierarchy, until all patients are combined in a unique group. This clustering procedure was based on Ward’s criterion^66^, which calculates the sum of within-cluster inertia for each partition. The choice of the optimal number of groups or clusters was guided on statistical criteria (the partition with the highest relative loss of inertia^67^; as well as interpretability and parsimony.

#### Interpretation of the typology of CTs

To interpret the types of CTs, various visual representations of the SSA were produced. Among them, State Distribution Plots show the distribution of states (proportion) for each time unit point, and Sequence Index Plots use line segments to show how individuals move from one state to another over time, each line representing an individual’s CT sequence. Once each patient was classified in a specific cluster (with similar CTs), covariables between groups were compared using the usual descriptive statistics (Chi-square test, Kruskal–Wallis test).

#### Sensitivity analyses

Several sensitivity analyses were performed to test the robustness of the results and their interpretation. First, different other distance measures were used: dynamic Hamming, generalized Hamming and optimal matching (with transition rate cost matrix)^64^. Afterward, another classification method was applied (k-means). To see if the results were sensitive to the priority order, priority order of states associated with the care providers was changed. Finally, the main analyses with different time units (“Months”, “Days”) were performed.

The SSA was performed using the TraMineR package in R^64,68,69^. All other analyses were performed using SAS 9.4.

## Supplementary information

Supplementary material

## Data Availability

The custom code that support the findings of this study are available on request from the corresponding author.
