# Supplementary material for: Multidimensional analysis of adult patients’ care trajectories before a first diagnosis of schizophrenia
Source: Schizophrenia (Heidelb). 2022 May 19;8(1):52. doi: 10.1038/s41537-022-00256-6 (PMC9261102; doi:10.1038/s41537-022-00256-6)

Supplementary Table 1: Healthcare use by dimension during the 2-year period before a first diagnosis of SCZ by the typology of Care Trajectories (CTs) (n=3712)

	**CT Type 1** Low HCU	**CT Type 2** Sharp 12-month HCU increase, mental Dis.	**CT Type 3** Mod. HCU, mental dis., primary care, community services	**CT Type 4** High HCU, mental dis., specialized care, psychiatrists	**CT Type 5** High HCU, Non-mental dis.	**TOTAL**
	**n=2881 (77.6%)**	**n=537 (14.5%)**	**n=98 (2.6%)**	**n=88 (2.4%)**	**n=108 (2.9%)**	**n=3712**
At least one HCU	2779 (96.5)	537 (100)	98 (100)	88 (100)	108 (100)	3610 (97.2)
At least one mental HCU	2377 (82.5)	518 (96.5)	-	-	-	3178 (85.6)
WHY						
At least one diagnosis of psychosis	1264 (43.9)	324 (60.3)	54 (55.1)	40 (45.4)	59 (54.6)	1554 (41.9)
At least one diagnosis of mood disorder	1250 (43.4)	319 (59.4)	64 (65.3)	79 (89.8)	46 (42.6)	1778 (47.9)
At least one diagnosis of anxiety	1000 (34.7)	251 (46.7)	52 (53.1)	58 (65.9)	39 (36.1)	1407 (37.9)
At least one diagnosis of another mental dis.	1227 (42.6)	334 (62.2)	72 (73.5)	60 (68.2)	88 (81.5)	1941 (52.3)
At least one diagnosis of non-mental dis.	2584 (89.7)	-	-	-	-	3386 (91.2)
WHERE						
At least one hospitalization	1072 (37.2)	355 (66.1)	55 (56.1)	74 (84.1)	83 (76.8)	1639 (44.2)
At least one ED visit	2050 (71.2)	449 (83.6)	77 (78.6)	79 (89.8)	98 (90.7)	2753 (74.2)
At least one visit to an outpatient clinic	1763 (61.2)	414 (77.1)	90 (91.8)	-	-	2449 (66.0)
At least one visit to a primary care clinic	2228 (77.3)	443 (82.5)	87 (88.8)	81 (92.0)	101 (93.5)	2940 (79.2)
At least one visit to a CLSC	1470 (51.0)	382 (71.1)	98 (100)	72 (81.8)	100 (92.6)	2122 (57.2)
WHICH						
At least one visit to a psychiatrist	1877 (65.2)	450 (83.8)	78 (79.6)	88 (100)	75 (69.4)	2568 (69.2)
At least one visit to another MD specialist	1893 (65.7)	407 (75.8)	87 (88.8)	77 (87.5)	103 (95.4)	2567 (69.2)
At least one visit to a GP	2498 (86.7)	487 (90.7)	-	-	-	3270 (88.1)
At least one visit to a nurse at CLSC	1008 (35.0)	269 (50.1)	80 (81.6)	53 (60.2)	91 (84.3)	1501 (40.4)
At least one visit to another worker at CLSC	782 (27.1)	283 (52.7)	93 (94.9)	54 (61.4)	86 (79.6)	1298 (35.0)

Supplementary Figure 1. Multidimensional state sequence analysis (adapted from Vanasse et al., 2020)

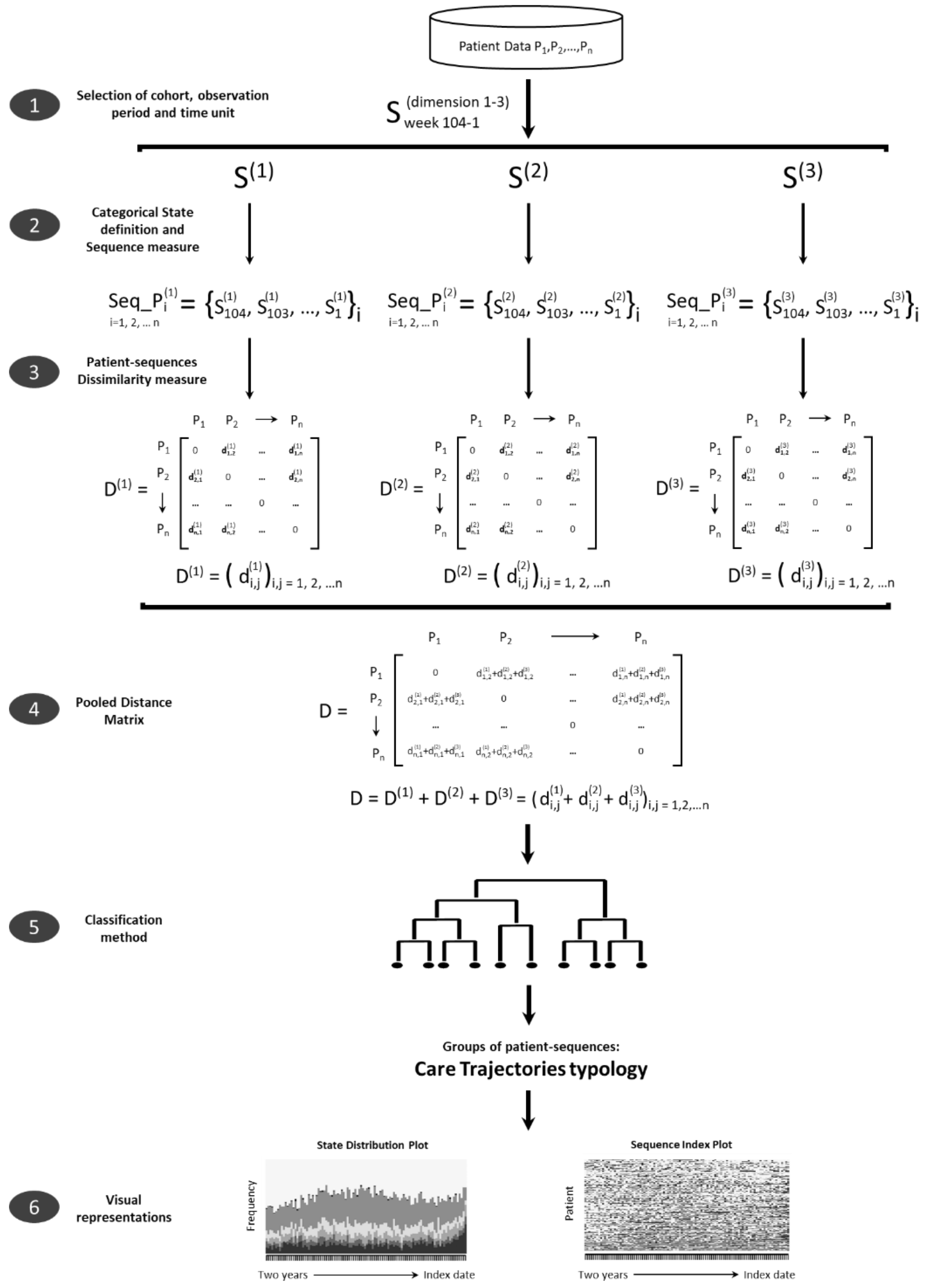

Supplementary Figure 2. Sensitivity analysis: State Distribution Plots of the typology of Care Trajectories (CTs) by dimension (why, where and which) using dynamic Hamming as the distance measure and HCA as the clustering method

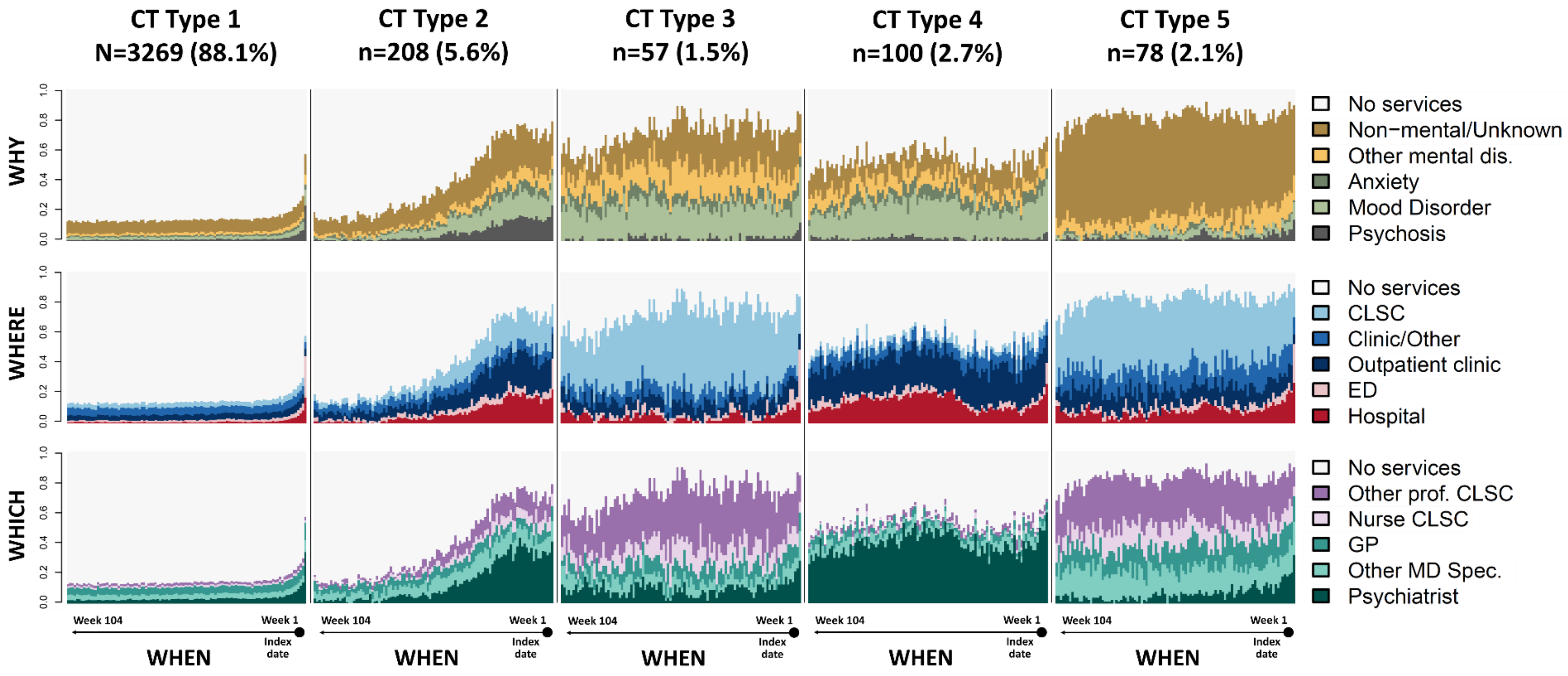

Supplementary Figure 3. Sensitivity analysis: State Distribution Plots of the typology of Care Trajectories (CTs) by dimension (why, where and which) using generalized Hamming as the distance measure and HCA as the clustering method

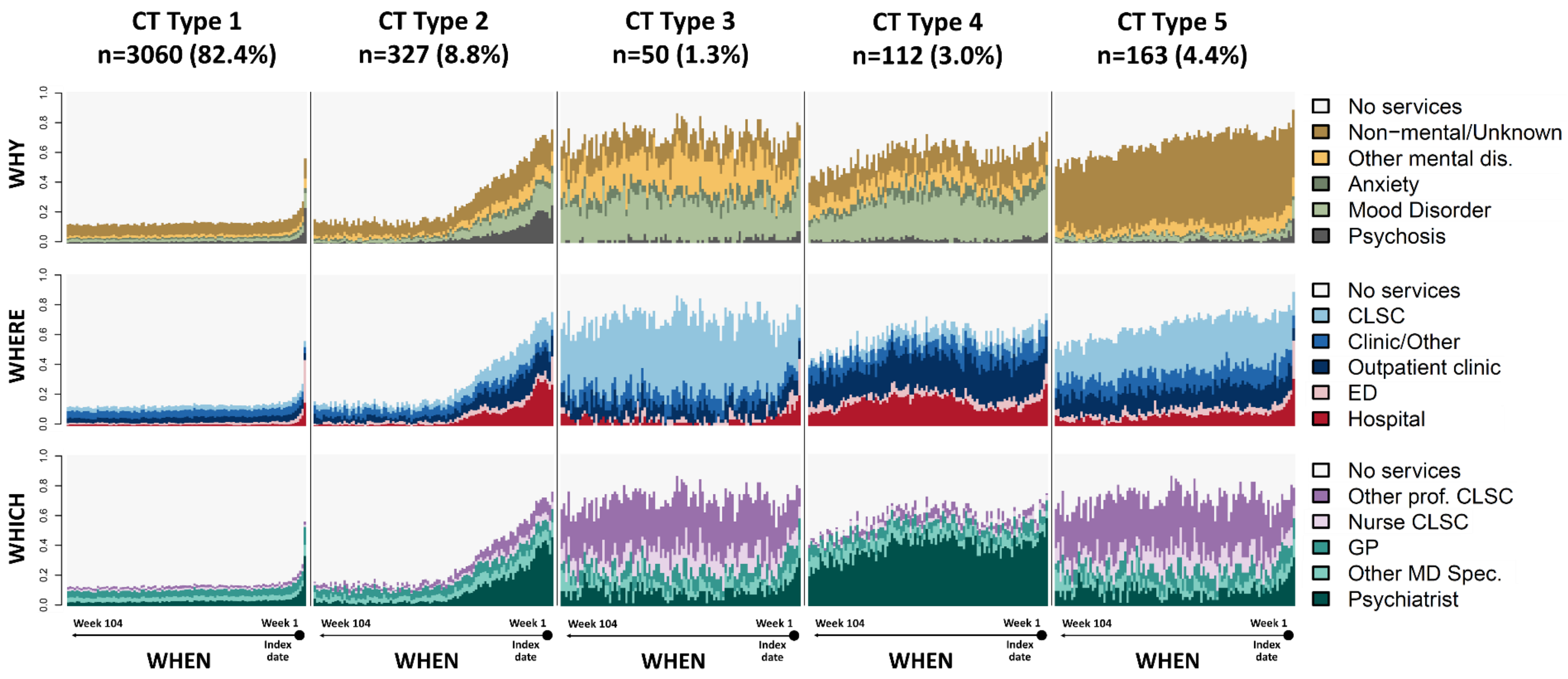

Supplementary Figure 4. Sensitivity analysis: State Distribution Plots of the typology of Care Trajectories (CTs) by dimension (why, where and which) using optimal matching as the distance measure and HCA as the clustering method

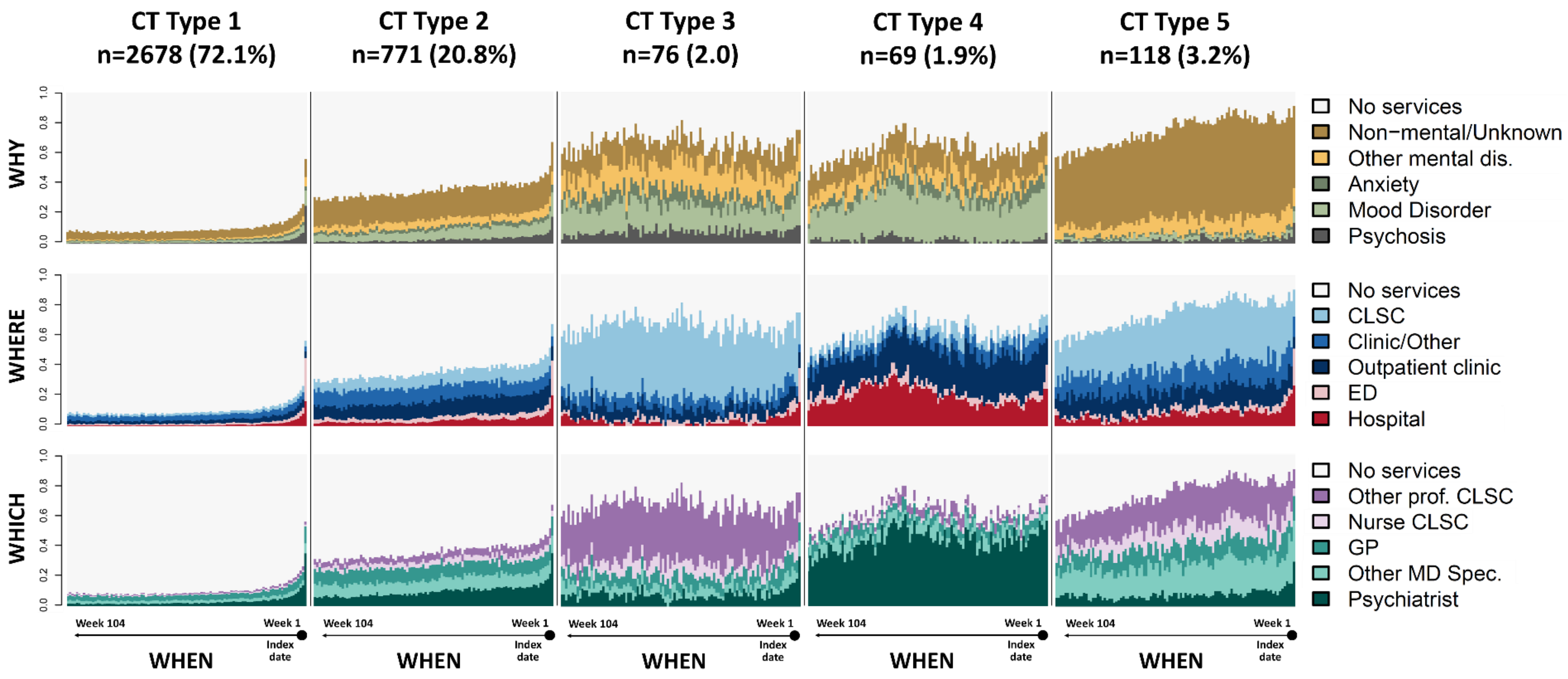

Supplementary Figure 5. Sensitivity analysis: State Distribution Plots of the typology of Care Trajectories (CTs) by dimension (why, where and which) using simple Hamming as the distance measure and k-means as the clustering method

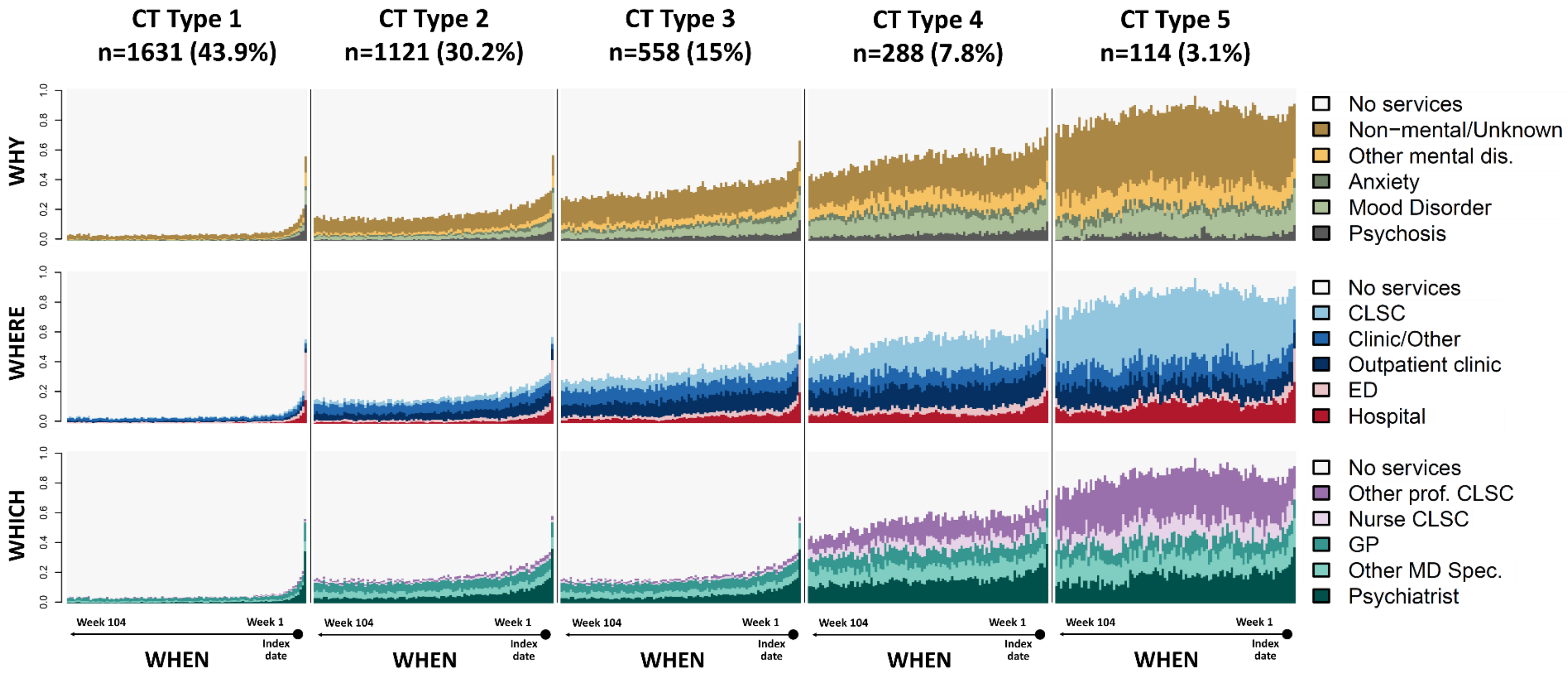

Supplementary Figure 6. Sensitivity analysis: State Distribution Plots of the typology of Care Trajectories (CTs) by dimension (why, where and which) changing the priority order in the which dimension (GP before Other MD Specialist)

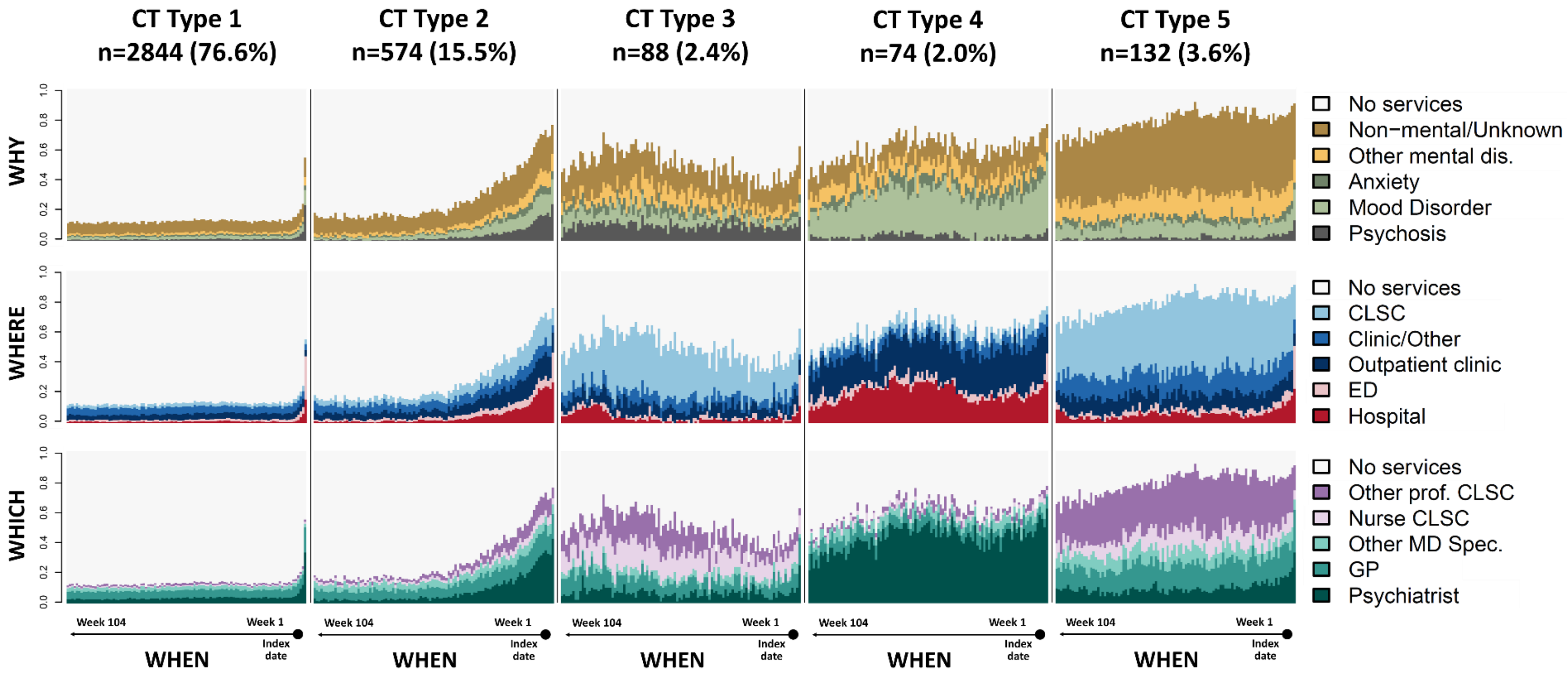

Supplementary Figure 7. Sensitivity analysis: State Distribution Plots of the typology of Care Trajectories (CTs) by dimension (why, where and which) using months as the time unit

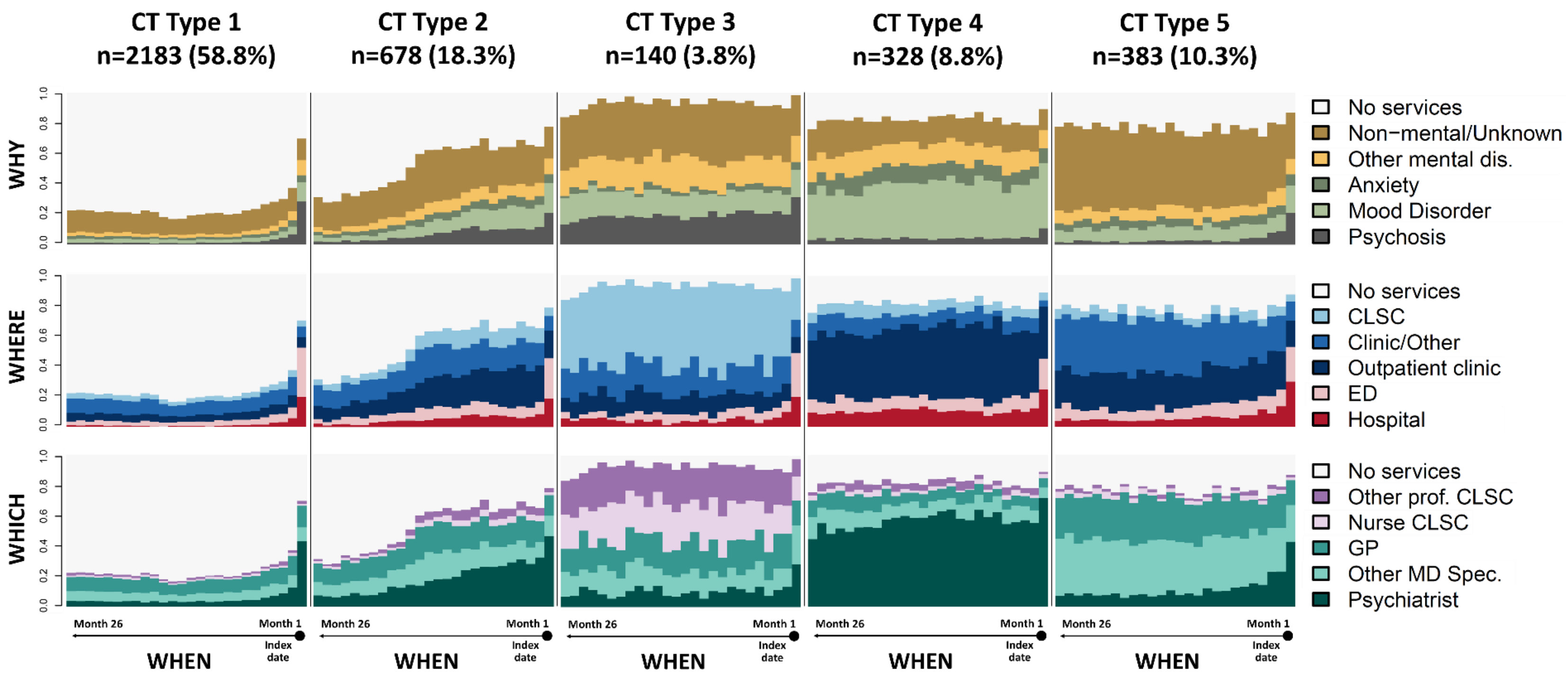

Supplementary Figure 8. Sensitivity analysis: State Distribution Plots of the typology of Care Trajectories (CTs) by dimension (why, where and which) using days as the time unit

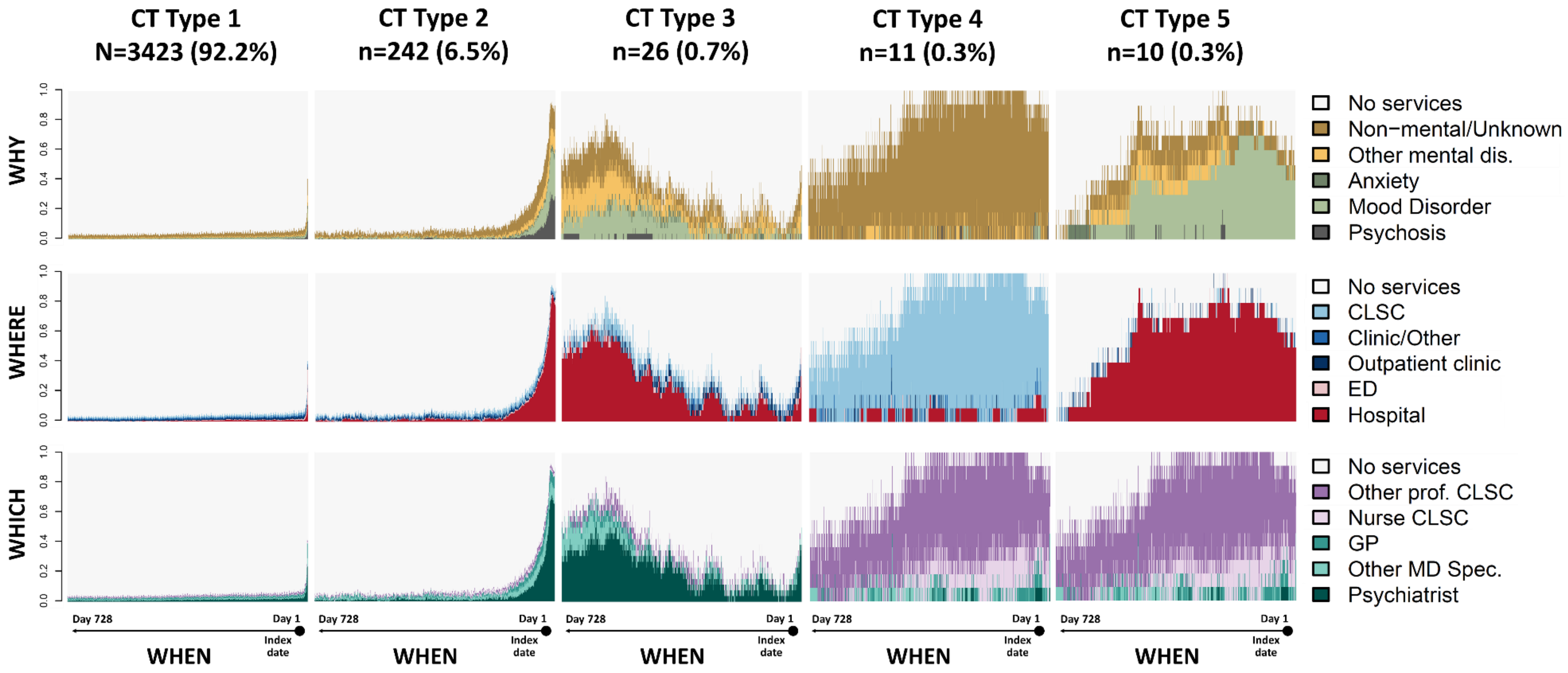

Supplement: Supplementary file 1 — Supplementary material [file 41537_2022_256_MOESM1_ESM.pdf]
